# Intestinal obstruction induced by a giant incarcerated Spigelian hernia. Case report and review of the literature

**DOI:** 10.1590/S1516-31802005000300012

**Published:** 2005-05-02

**Authors:** Edson Augusto Ribeiro, Samuel Martins Moreira

**Keywords:** Hernia, Acute abdomen, Intestinal obstruction, Tomography, Surgery, Hérnia, Obstrução intestinal, Tomografia, Abdome agudo, Cirurgia

## Abstract

**CONTEXT:**

Spigelian hernia is an uncommon spontaneous lateral ventral hernia with an incarceration ratio of around 20%. However, complications such as intestinal obstruction are extremely rare. We report on a case of giant incarcerated Spigelian hernia with a clinical condition of complete intestinal obstruction that was treated using prosthetic polypropylene mesh.

**CASE REPORT:**

A 72-year-old woman was admitted to the emergency department complaining of diffuse abdominal pain. Abdominal examination revealed a firm 10 × 10 cm tender mass in the lower left quadrant, without surrounding cellulite or tenderness. Plain abdominal radiographs displayed the formation of levels, thus indicating the existence of intestinal obstruction. An abdominal computed tomography scan clearly showed a fluid and air-filled mass in the soft tissue area of the lower left-side abdominal wall. Spigelian incarcerated hernia was diagnosed and the patient underwent emergency surgical repair by means of local incision. The large defect in the abdominal wall was closed up as successive anatomical layers, and a prosthetic polypropylene mesh was set into the lateral aspect of the rectus sheath. The postoperative course was uneventful and the patient was discharged on the seventh postoperative day.

## RESUMO

**CONTEXTO:**

A hérnia de Spiegel é uma rara hérnia da parede abdominal com uma taxa de encarceramento de cerca de 20%. Entretanto, as complicações relacionadas ao encarceramento, como a obstrução intestinal, são extremamente raras. Nós apresentamos um caso de abdome agudo obstrutivo induzido por uma hérnia Spiegel gigante tratada cirurgicamente com a colocação de uma prótese de polipropileno.

**RELATO DO CASO:**

Mulher de 72 anos foi admitida no Serviço de Emergência com queixa de dores abdominais. Ao exame apresentava massa de consistência firme de 10 × 10 cm de extensão no quadrante inferior esquerdo do abdômen. A radiografia de abdome apresentava múltiplos níveis hidro-aéreos. A tomografia axial de abdômen revelava massa na parede abdominal com conteúdo liquido e gasoso no quadrante inferior esquerdo. A paciente foi então submetida à cirurgia, sendo o defeito na parede abdominal fechado por planos anatômicos, seguida da colocação de uma tela de polipropileno que foi fixada à borda lateral do músculo reto abdominal. O pós-operatório foi sem intercorrências, tendo a paciente recebido alta no sétimo dia pós-operatório.

## INTRODUCTION

Spigelian hernia is an uncommon spontaneous lateral ventral hernia and is rarely considered in the differential diagnosis of patients with abdominal pain.^1^ The incarceration ratio of this rare type of hernia at the time of surgery has been reported to be around 20%.^1,2^ However, complications, such as intestinal obstruction, are extremely rare. We report on a case of giant incarcerated Spigelian hernia with a clinical condition of complete intestinal obstruction that was treated using prosthetic polypropylene mesh.

## CASE REPORT

A 72-year-old woman was admitted to the emergency department complaining of diffuse abdominal pain that she had had for two days. Over that period, she had developed a lower left quadrant abdominal mass. She had no history of nausea or vomiting. On admission, the patient was in good clinical condition, without fever and with a heart rate of 88 beats/min and blood pressure of 120 × 95 mmHg.

Abdominal examination revealed a distended abdomen with a firm 10 × 10 cm tender mass in the lower left quadrant, without surrounding cellulite or tenderness. The bowel sounds were above normal levels. Rectal examination revealed that the ampulla was empty. The leukocyte count was 10,800/ mm^3^, with normal differential. Other laboratory data, including hemoglobin, bilirubin, alkaline phosphatase, amylase and glucose concentrations, were normal.

**Figure 1:**
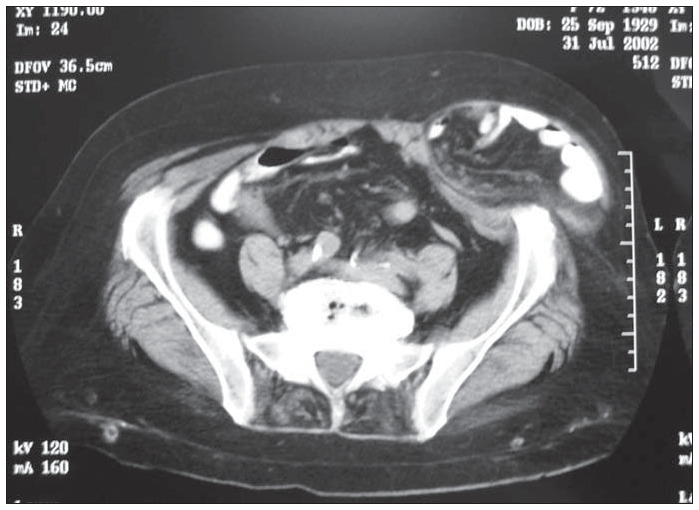
Abdominal computed tomography scan in a 72-year-old woman with a Spiegel incarcerated hernia: fluid and air-filled mass in the soft tissue area of the lower left-side abdominal wall.

**Figure 2:**
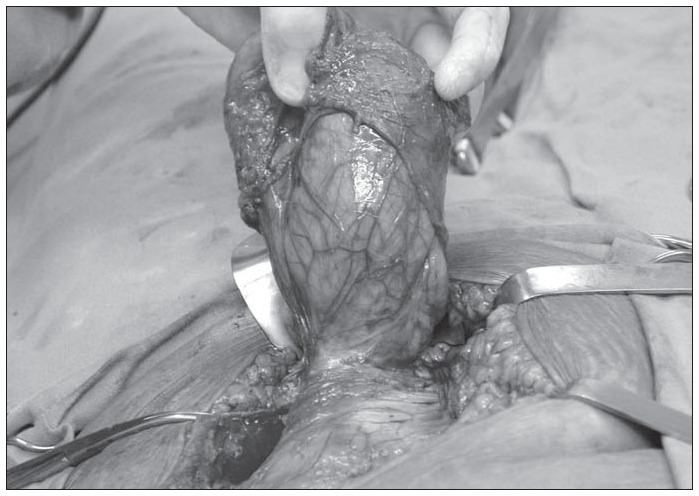
Intra-operative findings: identification of hernial sac in a 72-year-old woman with intestinal obstruction.

Plain abdominal radiographs displayed the formation of levels, thus indicating the existence of intestinal obstruction. An abdominal computed tomography scan clearly showed a fluid and air-filled mass in the soft tissue area of the lower leftside abdominal wall (Figure 1). Spigelian incarcerated hernia was diagnosed and the patient underwent emergency surgical repair. A paramedian incision was made in the lower left quadrant, with dissection of subcutaneous adipose tissue. Following this, the hernia sac was clearly recognized and opened; no ischemic changes were detected in either the intestine or the omentum. Both organs were then reduced to the abdominal cavity. After complete dissection, the large defect was closed up as successive anatomical layers, and a prosthetic polypropylene mesh was set into the lateral aspect of the rectus sheath, thereby covering the abdominal wall defect (Figure 2).

The postoperative course was uneventful and the patient was discharged on the seventh postoperative day. She is at present in a satisfactory condition and remains under observation as an outpatient, without any signs of hernia recurrence.

## DISCUSSION

Spigelian hernia occurs through congenital or acquired defects in the Spigelian fascia. This is the area of the transversus abdominis aponeurosis, lateral to the edge of the rectus muscle but medial to the Spigelian line, which is the point of transition of the transversus abdominis muscle to its aponeurotic tendon.^1,3^ Klinklosch, in 1764, was the first to describe a Spigelian hernia. However, it was only in 1976 that Spangen described in detail the anatomic aspects of Spigelian hernias in his thesis.^4^

Eight years later, this same author reviewed the published material on Spigelian hernias. At that time, 744 patients had required surgery for this condition. Although the proportion of the patients requiring emergency surgery was not reported, only 21% of all cases were classified as incarcerated at the time of surgery. The hernia sac usually contains the greater omentum. However, involvement of other organs has been reported, including the small intestine, colon, stomach, gallbladder, Meckel's diverticulum, appendix, ovaries and testes.^1-3^

Although cases of Spigelian hernia in infants and children have previously been described, these hernias have mainly occurred among adults between 40 and 70 years of age, and generally in obese females who have undergone parturition several times. Many reports on Spigelian hernias have emphasized the difficulty in making the diagnosis, for the following reasons: (1) the nonspecific variety of symptoms, (2) their small size, (3) the intramural location of the hernia (usually located between different muscle layers), and (4) the non-diagnostic findings on plain abdominal radiographs.^1,3^

Although there is a long list of possibilities regarding the differential diagnosis of superficial lesions in the abdominal wall, the most common diseases that mimic Spigelian hernia include rectus sheath hematoma, abdominal wall abscess and seroma. Less commonly, other entities such as fibroma, lipoma, sarcoma and hemangioma may be found.

The diagnostic procedures are mainly aimed at demonstrating a hernial orifice or sac. Plain abdominal radiographs and gastrointestinal tract studies using barium sulfate are diagnostic only if the bowel has herniated through the defect and appropriate oblique views are obtained.

Spangen, in 1984, was the first to use ultrasonic scanning for the diagnosis of Spigelian hernia. This approach has gained wider acceptance recently. According to Spangen, rapid and accurate diagnosis can be made by carefully scanning the abdominal wall to demonstrate the discontinuity in the echo line from the aponeurosis caused by a hernial orifice in the Spigelian fascia, at the level of the palpable mass or point of tenderness.^1,5^

Isolated case reports have demonstrated that computerized tomography (CT) scanning using closely spaced slices through a limited area may reveal the hernial orifice in the Spigelian fascia. However, Spangen stated in one of his articles that ultrasonography and CT scans probably have the same sensitivity for demonstrating the hernial orifice in the Spigelian aponeurosis.^1,3^

Nonetheless, most authors have found that many Spigelian hernias may remain undiagnosed until laparotomy is performed. Weiss et al. reported, in a series of 178 patients, that the correct preoperative diagnosis was made only in 92 cases (51.7%).^1,3^

Although uncommon, Spigelian hernias account for over 2% of cases undergoing emergency surgery for abdominal wall hernia. The treatment of this condition is always surgical, and typically has excellent results. In a review article published in 1989, only five recurrences after surgical treatment were reported out of a total of 876 patients (0.7%).^1^ Recently, laparoscopic management of this rare condition was described with very good results, and with the particular advantage of being able to treat concomitant intra-abdominal surgical affections as described by Fisher in 1994.^6^

This case report demonstrates the need to remain alert regarding the possibility that this rare entity is present. Detailed physical examination and choosing appropriate diagnostic procedures assist in achieving correct preoperative diagnosis, thereby contributing towards an adequate surgical approach.
